# Editorial del XLV Congreso de la SEMFYC

**DOI:** 10.1016/j.aprim.2026.103453

**Published:** 2026-02-02

**Authors:** Maria del Pino Calderin Morales, Ana Isabel Gonzalez González, Alberto Cotillas Rodero

**Affiliations:** a Comité Organizador y científico; b Comité Organizador y científico; c Presidente somamfyc

XLV Congreso de la SEMFYC – Madrid, 13-15 de noviembre de 2025**

El XLV Congreso de la Sociedad Española de Medicina de Familia y Comunitaria (semFYC), celebrado en Madrid del 13 al 15 de noviembre de 2025, ha sido todo un éxito desde el punto de vista científico, profesional y humano. Durante las tres jornadas, 2000 profesionales procedentes de todas las comunidades autónomas se reunieron para compartir conocimiento, experiencias y reflexiones, reafirmando la vitalidad, diversidad y compromiso de nuestra especialidad.

Nuestro lema «Somos agua, somos muro, somos fuego, somos Medicina de Familia y Comunitaria» ha guiado el espíritu del Congreso como una expresión simbólica y práctica de lo que somos: capacidad de adaptación, solidez profesional y compromiso permanente con la transformación de la Atención Primaria y con la salud de la comunidad.

El Congreso se inauguró con un acto que integró ciencia, arte, emociones y comunidad, amenizado con mucha sensibilidad. «La sala 13» cuenta con un guión elaborado por el PA-AP (prescripción de arte por atención primaria), **integrado a su vez por** María José Álvarez Pasquín, Alberto López García Franco, Javier Gómez Marco y Andrés Alex Lis Díaz, cuatro voces de la medicina pertenecientes a SoMaMFyC, que se atrevieron a recetar belleza, ritmo y emoción. Contaron con la colaboración de la violinista Judith Mateos, creando un espacio de apertura simbólica, que conectó a las personas asistentes con la dimensión más humana, creativa y social de la Medicina de Familia y Comunitaria.

Este inicio marcó el tono del Congreso, invitando a una participación consciente y a una mirada integradora del cuidado, donde el conocimiento científico convive con la escucha, la emoción y el vínculo terapéutico.

El programa reflejó la complejidad el día a día de la Medicina de Familia y Comunitaria, a través de una amplia variedad de actividades: talleres clínicos y prácticos para el día a día de la consulta, mesas de actualización, espacios de salud comunitaria, actividades de innovación docente, sesiones de investigación y comunicación científica, encuentros sobre ética, cronicidad, salud mental, cuidados paliativos y habilidades comunicativas, así como iniciativas muy novedosas y creativas vinculadas al arte y la salud. Asimismo, se reservaron espacios específicos para los médicos residentes y estudiantes universitarios, favoreciendo su participación y su desarrollo profesional.

En este contexto diverso, la investigación ocupó un lugar central como eje transformador de la Medicina Familiar y Comunitaria no solo como generadora de conocimiento, sino como herramienta clave para transformar la práctica clínica, reducir inequidades y fortalecer el sistema sanitario desde la atención primaria. A lo largo del Congreso se puso de manifiesto la consolidación de una investigación estrechamente vinculada a la realidad asistencial, orientada a problemas relevantes para la población y comprometida con la mejora de los resultados en salud. El congreso reflejó una clara evolución hacia enfoques metodológicos más integradores, capaces de abordar la complejidad de la multimorbilidad, la cronicidad y los determinantes sociales de la salud, incorporando de forma creciente el uso de datos en vida real, la investigación participativa y la evaluación de intervenciones complejas. Contar con la presencia de Raquel Yotti, comisionada del PERTE de Vanguardia, en el foro de investigación reforzó el compromiso de la medicina de familia hacia la investigación y la generación de conocimiento.

Este encuentro subraya, además, la necesidad de seguir reforzando las capacidades estructurales que sostienen la investigación en Medicina Familiar y Comunitaria —tiempo protegido, formación, redes colaborativas y apoyo institucional— como condición imprescindible para avanzar hacia un sistema sanitario más equitativo, sostenible y centrado en las personas. La Atención Primaria se reafirma, así como un espacio privilegiado para generar evidencia útil, aplicable y alineada con las necesidades reales de pacientes y profesionales.

La diversidad del programa diseñado para este XLV Congreso, no solo da cuenta de la amplitud de nuestra especialidad, sino también de su capacidad para integrar ciencia, humanismo, innovación y compromiso social en un marco formativo sólido y coherente.

El trabajo coordinado y meditado gracias a todas las aportaciones de los socios y de los GdT de semFYC y federadas, fueron claves para el Comité Científico y el Comité Organizador, que actuaron como una estructura cohesionada y complementaria, garantizando la coherencia, calidad y pertinencia de los contenidos y una clara apuesta por recuperar los espacios de actualización y debate sobre contenidos clínicos como herramientas muy útiles y necesarias, especialmente para los médicos residentes y jóvenes médicos de familia, para poder incorporar a la consulta del día a día.

La colaboración entre ambos equipos fue clave para que cada actividad alcanzara sus objetivos formativos y participativos, consolidando el Congreso como un espacio de referencia para la formación continua, la investigación y el desarrollo profesional en Medicina de Familia y Comunitaria.

Además, esto no hubiera sido posible sin el esfuerzo realizado de los grupos de trabajo de la semFYC y de la SoMaMFyC, auténticos artífices del programa científico, cuya implicación, creatividad y rigor han sido esenciales para la calidad del Congreso.

A su vez contamos con médicos de familia de renombre internacional como Tomás Zapata desde la OMS Europa recordándonos la importancia de innovar y reinventarse, así como la participación de Minna Johansson, directora del Global Center for Sustainable Healthcare, Raquel Gómez Bravo, co-chair of the WONCA Special Interest Group on Family Violence; Sara Ares miembro del Wonca Europe Working Party on Policy Advocacy y representante de semFYC en el EGPRN (European General Practice Research Networ); mostrando cómo la semFYC **lleva la evidencia y la experiencia de los médicos de familia a los responsables de decisiones**, para impulsar políticas que mejoren la Atención Primaria y garanticen un acceso equitativo y sostenible a la salud.

La participación de Pilar Astier, presidenta electa de WONCA World, y Paula Sala, presidenta electa del European Young Family Doctors Movement (EYFDM), simbolizan el reconocimiento internacional del liderazgo de semFYC como adalid de la Medicina de Familia y Comunitaria.

El acto de clausura mostró de una forma amable y divertida todas las impresiones y contenidos del congreso mediante el telediario semFYC News, conducido por Nuria Jiménez y Antonio Cabrera. La entrega de premios, por su parte, fue vehiculizada por el **GdT Salud basado en emociones de Somamfy**c, formado por Paloma Henares, Jorge Juan Prada, María del Carmen Álvarez, Hanz Carlos Alache, Francisco Abellán y Javier Dols. Con su presentación «El Retorno del resi» nos mostraron a través de las emociones los valores de nuestra especialidad y le dieron un toque de humor a esta clausura, ofreciéndonos un cierre reflexivo y coherente con los valores de la Medicina de Familia y Comunitaria.

Deseamos expresar un reconocimiento muy especial a las 2000 asistentes, procedentes de todo el país (junto a algún visitante internacional). Vuestra participación, la diversidad de miradas y el compromiso, han dado sentido al esfuerzo colectivo que supone un Congreso de estas características. Vosotros sois quienes transformáis el programa en comunidad, el conocimiento en intercambio y el encuentro en crecimiento compartido.

Porque somos agua, que fluye, se adapta y acompaña.

Somos muro, que sostiene y defiende la Atención Primaria.

Somos fuego, que impulsa, inspira y transforma.

Somos Medicina de Familia y Comunitaria, este Congreso ha contribuido a fortalecer, una vez más, nuestro compromiso colectivo con las personas, las comunidades y el sistema sanitario.

